# Enhancement of Bagasse Paper Properties for Sustainable Packaging via Bentonite and Alginate Loading in Shellac coatings

**DOI:** 10.1038/s41598-025-22179-9

**Published:** 2025-11-11

**Authors:** Salah A. A. Mohamed, Mohamed El-Sakhawy, Mohamed A. Diab

**Affiliations:** 1 Packaging Materials Department, National Research Centre, 33 El-Bohouth Str., Dokki, P.O. 12622, Giza, Egypt; 2 Cellulose and Paper Department, National Research Centre, 33 El-Bohouth Str., Dokki, P.O. 12622, Giza, Egypt

**Keywords:** Sodium alginate, Shellac, Bentonite, Modified bagasse, Paper-based packaging materials, Chemical safety, Green chemistry, Materials chemistry

## Abstract

This study addresses a key limitation in sustainable packaging; the inadequate mechanical strength and barrier properties of unmodified paper-based materials, particularly those made from agricultural residues like bagasse. Although natural biopolymers such as shellac, alginate, and bentonite have shown individual promise in improving paper properties, their combined effects and potential synergistic interactions remain underexplored. Here, we investigate how incorporating bentonite and alginate into a shellac matrix improves the mechanical, hydrophobic, and air-barrier characteristics of bagasse paper sheets. Three composite formulations were prepared; the first by mixing a 2% alginate solution with 1–5% shellac; the second by mixing a 1% bentonite solution with 0.5–3.5% shellac; and the third by combining 1% bentonite with 2% alginate and 1–5% shellac. These formulations were applied to bagasse paper via a simple dip-coat and oven-dry protocol. Scanning Electron Microscopy (SEM) confirmed the formation of a uniform, continuous macromolecular network that facilitated enhanced surface coverage and cohesion. Results revealed that the addition of alginate and bentonite significantly improved both mechanical and barrier properties. For instance, a 2% shellac formulation when combined with 2% alginate or with a 2% alginate/1% bentonite mixture, eliminated air permeability and doubled the burst strength. These enhancements are attributed to increased hydrogen bonding and ionic interactions among the components, which enhance composite density and structural integrity. The most effective formulation (2% alginate + 3% shellac) improved tensile strength by 129% and elongation by 103%, demonstrating a desirable balance of rigidity and flexibility. Consequently, the modified bagasse paper sheets exhibit superior tensile strength, reduced air permeability, improved water vapor permeability (WVP), enhanced thermal stability, and increased biological activity compared to untreated sheets, single-component (shellac-only) and binary (shellac–alginate or shellac–bentonite) systems previously reported. These findings introduce a novel ternary shellac/alginate/bentonite bio-based composite coating system that significantly enhances the functional performance of bagasse paper. This system represents a viable, eco-friendly solution for sustainable packaging applications requiring moisture resistance, mechanical durability, and biodegradability.

## Introduction

Packaging plays a crucial role in transporting food safely, extending shelf life, and protecting contents from microbial contamination and physical degradation. Plastic materials dominate the food packaging industry due to their excellent mechanical strength, low cost, and ease of processing. However, increasing concerns about environmental sustainability and plastic pollution have driven the need for eco-friendly, cost-effective, and high-performance alternatives that preserve food quality, and enhance barrier and mechanical properties^1^. Recent studies have explored multi-functional coatings for paper packaging. For instance, polyvinyl alcohol (PVA) composite with zinc oxide and natural extracts showed improved tensile strength and hydrophobicity^2^. Another study demonstrated that TEMPO-oxidized bacterial cellulose films could be enhanced through hot vapor treatments, improving mechanical and water-resistance performance^3^. Similarly, novel antimicrobial nano-coatings like erythrosine-dialdehyde cellulose nanocrystals have been developed to address the paper’s lack of inherent antimicrobial activity^4^.

Among the various biodegradable coating materials, shellac and alginate are promising natural biopolymers. Shellac is a biodegradable, edible, renewable, and non-toxic resin with excellent water barrier properties. However, it shows poor oxygen barrier capacity and limited oxidative stability due to polymerization^5^. Conversely, alginate, a natural polysaccharide extracted from brown seaweed, provides good oxygen barrier properties, film-forming capabilities, and resistance to oils and fats. Nonetheless, alginate-based films are typically brittle, hygroscopic, and mechanically weak, which limits their application^6^. Therefore, combining alginate and shellac is expected to complement and improve each other’s deficiencies^5^. A new method for improving nut preservation and reducing aflatoxin contamination is investigated in this work: coatings made of chili and garlic oleoresin nanoparticles. Bioactive content and oxidative stability were used to describe oleoresins. 4.5% Arabic gum and 1.5% carboxymethyl cellulose were used to create nanoparticle membranes, which were then reinforced with either alginate or maltodextrin. While garlic oleoresin increased the mechanical resilience of alginate membranes from 6.05 MPa to 27.18 MPa, the results showed that chili oleoresin increased the tensile strength of maltodextrin-based membranes from 1.71 MPa to 13.99 MPa^7^.

To address the weaknesses of these polymers, recent studies have attempted to combine shellac and alginate into composite coatings, achieving moderate improvements in film performance. For example, shellac-alginate/carrageenan films reinforced with cellulose nanocrystals (CNC) showed excellent properties to extend the storage time of chicken breast and cherry tomatoes in the food storage experiment^8^. Nanoclays, a class of reinforcement materials available in abundance with nanoscale dimensions, are comprised of layered silicates. Commonly, layer silicates are held together by weak van der Waals forces between their layers, while the strong bonds within the layers themselves is mainly ionic and covalent^9,10^.

Biopolymer matrix reinforced with nanoclay, as filler, could be used to meet the food packaging demand and the environmental. To further improve the structural and barrier performance of biopolymer coatings, nanoclays such as bentonite have been employed. Nanoclays are nanoscale layered silicates that enhance mechanical strength, thermal resistance, and gas/water vapor barrier properties due to their high aspect ratio and twisting path effect^11^. Bentonite, in particular, consists of alternating layers of silica and aluminum hydroxide and has been used in food and pharmaceutical applications. In biopolymer matrices, bentonite acts as a reinforcing filler, significantly improving coating performance and the biopolymer properties of films for food packaging applications^12,13^.

Most studies have focused on single-component films such as shellac^14^ and alginate^15^, and binary systems like shellac-clay^14^ or alginate-shellac^5^. However, these coatings typically offer partial enhancements in water vapor resistance or tensile strength, meanwhile, the synergistic effects of combining shellac, sodium alginate, and bentonite in a single coating system have not been systematically explored, especially on agro-residue substrates such as bagasse paper. This knowledge gap is particularly acute for bagasse paper whose full valorization remains limited by sub-optimal barrier and mechanical properties. When the PVA/SC/curcumin/shellac formulation was applied to the NWC fabric sample, the following outcomes were observed: (a) the treated fabric’s gel fraction increased along with its swelling characteristics decreased. In comparison to other treated formulations, the treated fabric exhibited the following benefits: (b) improved antimicrobial activity against filamentous fungus (*Aspergillus niger*), pathogenic yeast (*Candida albicans*), and Gram-positive and Gram-negative bacteria (*Staphylococcus aureus* and *Escherichia coli*); (c) reduced air permeability and increased tensile, Young’s, and burst strengths; and (d) the best water vapor transmission rate^16^.

This study introduces a novel ternary composite coating composed of shellac, sodium alginate, and bentonite, applied to bagasse paper sheets via a simple immersion-drying process. Shellac’s hydrophobic macromolecular network, alginate’s hydrogen-bonding and film-forming ability, and bentonite’s nano-barrier effects, which create a twisting path for gases and moisture, could overcome the limitations of single or binary coatings to enhance tensile and burst strength and improve air permeability, water vapor permeability, and thermal stability along with the antimicrobial activity of the coated paper to assess its viability as an eco-friendly packaging material. The proposed coating offers a sustainable, biodegradable, and high-performance alternative to petroleum-based coatings currently used in packaging. The demonstrated improvements suggest strong potential for industrial scalability in food packaging, wrapping, or disposable containers, especially where moisture, mechanical integrity, and biological resistance are critical.

## Materials and methods

### Materials

Raw bagasse was provided by the Egyptian Quena Paper Industry Company and examined using standard techniques for cellulose chemical analysis. In the laboratory, bagasse pulp was produced using soda pulping techniques.

Bentonite, whose mineralogical and chemical composition comprises mainly silica (SiO₂), alumina (Al₂O₃), and iron oxide (Fe₂O₃) in approximate amounts of 61 wt%, 18 wt%, and 2 wt% respectively^12^, and food-grade sodium alginate was purchased from Shanghai Chemical Reagent Co. (Shanghai, China). Commercial-grade shellac was used as received.

#### Preparation of the bagasse pulp

Bagasse pulp was produced by pulping bagasse straw with an aqueous sodium hydroxide solution at 150 °C for two hours. Following neutralization with water, the pulp was bleached using a sodium chlorite/acetic acid mixture. The chemical composition of the bleached pulp, Klason lignin, holocellulose, hemicelluloses, and ash content was determined according to standard methods. Other chemicals were of analytical grade and used as received.

#### Preparation of coated bagasse paper sheets with bentonite and alginate in a shellac matrix

Separate experiments involved dissolving 2 g of sodium alginate and 1 g of bentonite in 100 mL of water, and dissolving 5 g of shellac in 100 mL of alcohol with continuous stirring for 1 h.

**Fig. 1:**
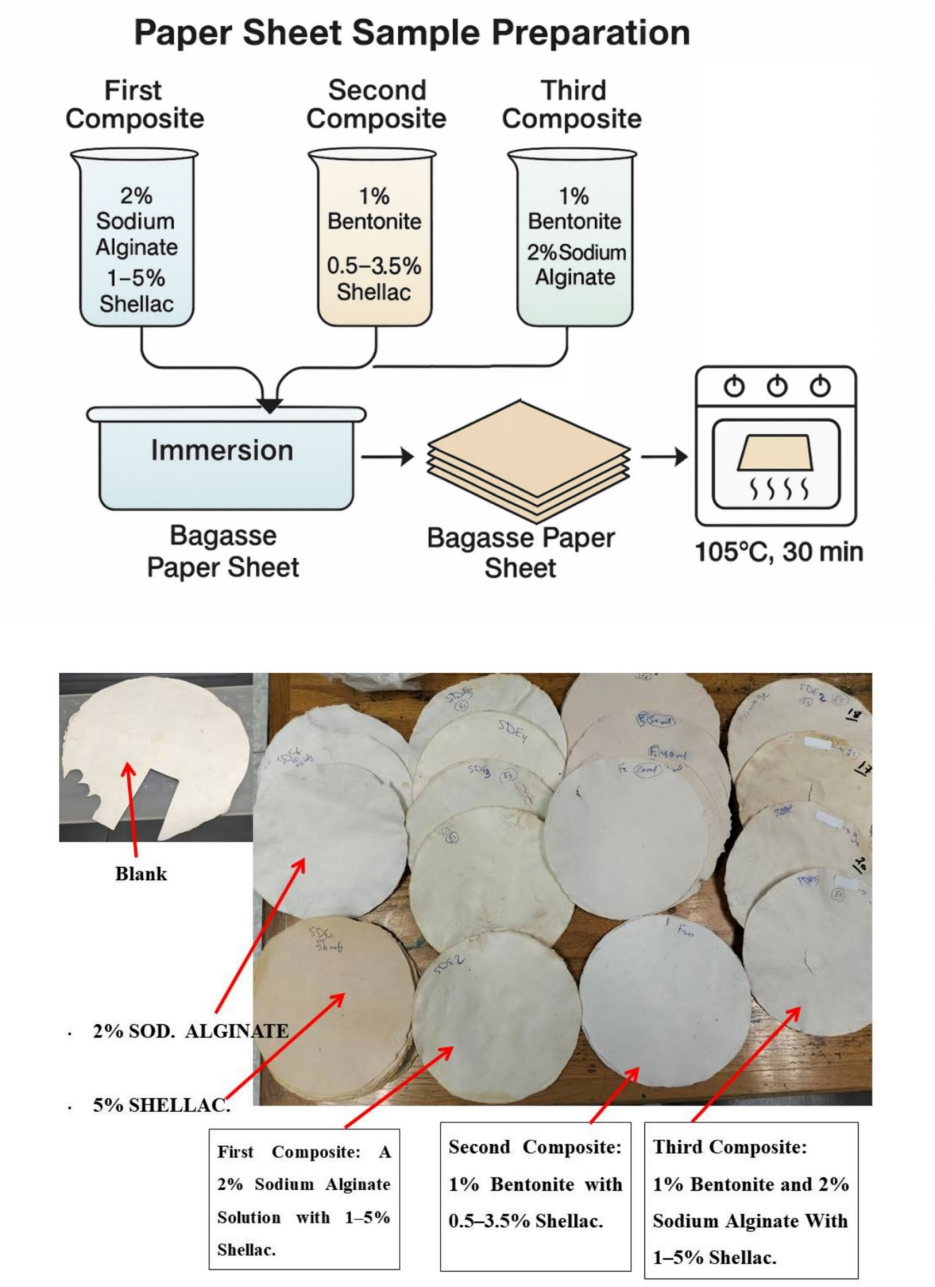
**T**he schematically diagram of sample preparation, and real samples image, (notice that the three composites depend on shellac).

This study evaluated the influence of incorporating bentonite and alginate into a shellac-based coating as a strengthening barrier for potential packaging applications using paper sheets. Three composite formulations were prepared and applied to the surface of bagasse paper sheets:

- *First Composite*: A 2% sodium alginate solution was mixed with 1–5% shellac.
- *Second Composite*: A 1% bentonite solution was combined with 0.5–3.5% shellac.
- *Third Composite*: A mixture of 1% bentonite and 2% sodium alginate was blended with 1–5% shellac. The schematical diagram of samples preparation, and real samples image, (notice that the three composites depend on shellac) were shown in Fig. 1.

### Scanning electron microscopy (SEM)

The microstructure of the coated paper sheets was imaged using a FEI Quanta 200 scanning electron microscope (FEI Company BV, Netherlands) at an acceleration voltage of 20 kV^16^.

### Thermogravimetric analysis (TGA)

The TG and DTG curves for the treated paper sheets were recorded simultaneously using a TA Instruments AC7/DX TGA7 (PerkinElmer). The experiments were conducted in a dynamic nitrogen atmosphere (20 mL/min) with a heating rate of 10 °C/min over a temperature range of 20–400 °C using platinum crucibles^7^.

### X-ray diffraction (XRD)

XRD analysis was performed using a Siemens D5000 diffractometer with CuKα (λ = 1.5406 Å) radiation, operating at 30 mA and 40 kV, to study the structural characteristics of the modified sheets^16^.

### Mechanical strength testing

Mechanical properties; including tensile, burst; was determined according to TAPPI standards. The average and standard deviation of the measurements were calculated based on five replicates for each sample. Tensile strength was measured in accordance with the TAPPI T494-06 standard method using a universal testing machine (LR10K; Lloyd Instruments, Fareham, UK) with a 100-N load cell and a constant crosshead speed of 2.5 cm/min^17^. Test specimens were strips 10 cm long and 15 mm wide with a 10 cm gauge length. While burst strength was measured using a Mullen tester (Chicopee, MA, USA) following TAPPI T403 om-10. The sheet thickness was measured using the TAPPI T411 om-97 standard method with a Käfer micrometer^18^.

### Barrier properties

Before testing, all samples were conditioned at 23 °C and 50% relative humidity (RH) for at least 24 h.

#### Air permeability

Air permeability is a critical property of packaging papers, as water vapor and oxygen can permeate through the material and degrade food quality. Measurements were conducted according to ISO 5636 using a Bendtsen smoothness and porosity tester (manufactured in Denmark by Andersson and Sorensen, Copenhagen). Air permeability was determined by measuring the airflow rate under standard pressure between the paper surface and two concentric annular metal rings applied to a sample area of 2 cm^2^ under ambient conditions with a 2.5 kPa vacuum. Each test was run for 5 min, and the average air permeability was calculated from at least five measurements^19,20^. This parameter reflects the degree of resistance of the paper web to airflow, which depends partially on the uniformity and porosity of the coating layer^7^.

#### Water vapor permeability (WVP)

The static water vapor permeability of the modified paper sheets was determined according to ASTM E96-95. Sheets were conditioned at 25 °C and 60% RH for 24 h before being used to hermetically seal aluminum cups containing 5 g of anhydrous calcium chloride. WVP was calculated from the slope of the mass gain versus the time curve, using the following formula:

$${\text{WVP }}\left( {{\text{g}}.{\text{m}}^{{ - {1}}} .{\text{s}}^{{ - {1}}} .{\text{Pa}}^{{ - {1}}} } \right)\, = \,\Delta {\text{m}}.{\text{ e }}/ \, [{\text{A}}. \, \Delta {\text{t}}. \, \Delta {\text{P}}]$$

where ∆m is the mass increase (in grams) of the CaCl_2_, A is the area of the modified paper sheet, and ∆t is the exposure time. e is the thickness of the modified paper sheet and ∆P is the partial water vapor pressure difference across the modified paper sheet specimen corresponding to 0–60% RH, i.e., 1875 Pa.

### Oil resistance test for paper

The oil resistance was measured according to the ASTM D722-93 method. In this study, olive-oil; one of the healthiest food oils and a key component of the Mediterranean diet due to its distinctive organoleptic characteristics, nutritional benefits, and cultural significance; was used for sample testing. A portion of the modified paper samples was conditioned at 25 °C and 50% relative humidity (RH). Next, the conditioned samples were placed in contact with a piece of white book paper, and 5 g of sand (with a specified particle size) saturated with a known quantity of oil containing a soluble red dye was placed on top. The time required for the oil to penetrate the modified samples and stain the book paper red was measured to the nearest 10 s, and the final value was calculated as the average of three measurements^6^.

### Contact angle measurement

The average contact angle of the prepared paper sheets was determined using a video contact angle system (KrÜss DSA 25B, Germany). The average surface hydrophilicity was obtained via applying water droplets on each paper sheet surface at five different locations. The surface of the sample under examination received an average of 250 μL of water. The test was carried out with the ambient temperature at 22 ± 1 ºC and the relative humidity at 65 ± 5%. At a 95% confidence level, the increased measurement uncertainty was ± 1° (coverage factor (k = 2))^21,22^.

### Antibacterial activity

The disc diffusion method was used to evaluate the antibacterial activity of the paper sheets fabric, and control (AATCC Test Method 147–2004). The diameter of the inhibitory zone (mm) was used to express the results. The antibacterial activity of the treated samples SDE(1–5), SDF(0–7) and SDG1-5, against Gram-negative bacteria ***Escherichia coli*** and Gram-positive bacteria ***Bacillus subtilis* (B)**. Nutrient agar plates were uniformly inoculated with 0.1 ml of 105–106 cells/ml for bacteria. Czapek-Dox agar plates were seeded with 0.1 ml (106 cells/ml) of the fungal inoculum in order to measure the antifungal activity. In samples of modified weave, treated discs (15 mm in diameter) were placed over the inoculation plates. Paper sheet-treated discs with a diameter of 15 mm were placed over the inoculation plates. To optimize diffusion, plates were then kept at a low temperature (4 °C) for two to four h. To encourage the organisms’ maximum growth, the plates were next incubated upright for 24 h at 37 °C for bacteria. The test agent’s antibacterial activity was determined by measuring the zone of inhibition’s diameter and expressing the result in millimeters (mm). Every experiment was run several times, and the mean value was noted^21,23^.

### Statistical analysis

Statistical analysis Duncan’s New Multiple Range test was performed to compare the means of the treatments using the MSTAT Computer Program (MSTAT Development Team, 1989). A fully randomized block design was used for the analysis of variance. Steel and Torrie (1980) conducted a statistical analysis of the^24,25^.

## Results and discussion

This study evaluated the influence of incorporating bentonite and alginate into a shellac-based coating as a strengthening barrier for potential packaging applications using paper sheets. Three composite formulations were prepared, as described in the experimental part, and applied to the surface of bagasse paper sheets as the following:

- *First Composite*: A 2% sodium alginate solution was mixed with 1–5% shellac.
- *Second Composite*: A 1% bentonite solution was combined with 0.5–3.5% shellac.
- *Third Composite*: A mixture of 1% bentonite and 2% sodium alginate was blended with 1–5% shellac. The results are summarized in Table 1.

**Table 1:** Characterization of the effect of adding shellac/alginate/bentonite on air permeability and burst strength of bagasse paper sheets.

Sample	Shellac (wt.%)	Burst strength (kg/cm^2^)	Air permeability (sec/100 mL)
Blank	0.00	2.15 ± 0.001	31.50 ± 1.470
SDE0	0.00	4.30 ± 0.101	Nil
SDE1	1.00	2.40 ± 0.002	Nil
SDE2	2.00	4.40 ± 0.003	Nil
SDE3	3.00	4.30 ± 0.100	Nil
SDE4	4.00	4.25 ± 0.002	Nil
SDE5	5.00	4.25 ± 0.000	Nil
LSD at 5%		0.16	
First composite: At [Alginate], 2%. Notice that: SDE0 is the paper sheet treated with alginate			
Blank	0.00	2.15 ± 0.001	31.50 ± 1.470
SDF0	0.00	2.65 ± 0.004	45.00 ± 2.202
SDF1	0.50	2.70 ± 0.002	65.00 ± 1.706
SDF2	1.00	2.95 ± 0.001	84.00 ± 2.504
SDF3	1.50	2.30 ± 0.000	87.00 ± 3.850
SDF4	2.00	2.95 ± 0.005	160.00 ± 2.540
SDF5	2.50	3.65 ± 0.004	155.00 ± 1.650
SDF6	3.00	3.30 ± 0.101	111.00 ± 2.120
SDF7	3.50	2.90 ± 0.006	106.00 ± 1.200
LSD at 5%		0.280	9.380
Second composite: At [Bentonite], 1%. Notice: SDF0 is paper sheet treated with 1% bentonite			
Blank	0.00	2.15 ± 0.001	31.50 ± 1.470
SDG1	0.00	4.30 ± 0.012	Nil
SDG2	1.00	4.25 ± 0.002	Nil
SDG3	2.00	4.30 ± 0.001	Nil
SDG4	3.00	4.10 ± 0.000	Nil
SDG5	4.00	4.20 ± 0.002	Nil
SDG6	5.00	4.40 ± 0.002	Nil
LSD at 5%		0.310	
Third composite: Note: All samples were prepared with fixed levels of 2% sodium alginate and 1% bentonite			

Treatment with 1% bentonite, 5% shellac, and 2% sodium alginate individually increased the burst strength by approximately 23.3%, 29.3%, and 100% (yielding burst strengths of 2.65, 2.8, and 4.3 kg/cm^2^, respectively) compared to the untreated sample (2.15 kg/cm^2^). In terms of air permeability, the 2% alginate treatment achieved a complete barrier (i.e., nil permeability), while treatments with 1% bentonite and 5% shellac resulted in values of 45 and 54 s, respectively (compared to 31.5 s for untreated sheets). Overall, the best improvements in both burst strength and air permeability were observed with 2% sodium alginate, followed by 5% shellac, and then 1% bentonite with the lowest enhancement.

### The first composite: shellac/alginate coating

#### Preparation and characterization

The effect of adding 0–5% shellac to a constant concentration of 2% sodium alginate on the mechanical and physical properties of bagasse paper sheets was investigated. As shown in Table 1, combining shellac with 2% sodium alginate significantly improved the burst strength compared to untreated paper sheets. The optimal burst strengths were achieved with 2% shellac, which produced improvement of approximately 104.65% (yielding burst strength of 4.4 kg/cm^2^,) relative to the 2.15 kg/cm^2^ observed in the untreated samples. The interaction between shellac and sodium alginate creates a composite matrix where shellac’s film-forming properties reinforce the alginate gel network, increasing mechanical strength and stability. Shellac’s ability to act as a natural polymer helps reduce air permeability and improves the surface smoothness, contributing to enhanced physical properties such as stiffness and durability^8^. Furthermore, all concentrations of shellac used in combination with 2% sodium alginate led to significant enhancements in air permeability. The untreated paper exhibited an air permeability value of 31.5 s, while the modified sheets showed no measurable air permeability. These improvements are likely attributable to the effective cross-linking between shellac and alginate, which creates a smoother and more compact film that adheres strongly to the cellulose surface^26^.

### The second composite: shellac/bentonite coating

#### Preparation and characterization

The burst strength and air permeability of paper sheets treated with various concentrations of shellac (0.5–3.5%) at a fixed 1% bentonite level were investigated, as summarized in Table 1. All shellac/bentonite combinations produced significant improvements in burst strength compared to untreated paper. The optimal results for burst strength were achieved at 2.5% shellac (yielding 3.65 kg/cm^2^, a 69.77% increase) and at 3% shellac (yielding 3.3 kg/cm^2^, a 53.49% increase), relative to the 2.15 kg/cm^2^ measured for the untreated sheets.

In addition, all shellac/bentonite treatments improved air permeability. The best air permeability results were recorded with 2% and 2.5% shellac additions, providing values of 407.94 s/100 mL (160 s) and 392.06 s/100 mL (155 s), respectively, compared to 31.5 s for the untreated sheets. These results are most likely due to the formation of a homogeneous and continuous film on the surface of the cellulose fibers, as observed by SEM (Fig. 8a). The synergistic interaction between shellac and bentonite acts as an effective cross-linker, yielding a smoother and more compact film than when either shellac or bentonite is used alone. The overall trend indicates that the combination of shellac and 1% bentonite significantly improved both burst strength and air permeability. An inverse relationship was observed; higher shellac concentrations, which result in thicker coatings, generally yielded lower air permeability values.

These findings suggest that blending shellac with either alginate or bentonite improves the mechanical and barrier properties of bagasse paper sheets. In particular, the shellac/alginate combinations yielded superior tensile and barrier performances, while the shellac/bentonite formulations also enhanced the material properties by forming a compact and continuous film on the cellulose surface^27^. This work demonstrates the promising potential of these modified bagasse paper sheets for future environmentally friendly packaging applications.

## The third composite

### Characterization of the effect of adding shellac/alginate/bentonite on the air permeability and burst strength properties of bagasse paper sheets

The influence of bentonite and alginate, incorporated into a shellac matrix, on the mechanical and hydrophobic properties of paper sheets was examined, focusing on air permeability and burst strength. In this study, paper sheets were treated with various concentrations of shellac (1–5%) while maintaining fixed concentrations of 2% sodium alginate and 1% bentonite. The resulting burst strength and air permeability values are presented in Table 1.

It is evident from the results that the addition of shellac to the constant formulation (2% sodium alginate and 1% bentonite) marginally affects the burst strength of the paper sheets compared to untreated samples. The best outcomes were observed at 2% and 5% shellac, which increased the burst strength by approximately 100% and 104.65% respectively (yielding values of 4.3 and 4.4 kg/cm2,) compared to 2.15 kg/cm2 for untreated sheets. Likewise, incorporating any concentration of shellac (ranging from 0 to 5%) with 2% sodium alginate and 1% bentonite eliminated air permeability, reducing it from 31.5 s for untreated specimens to zero, which is comparable to or better than values reported for conventional polyethylene-coated paperboard^28^, and for bio-based coatings^29^.

These results may be attributed to the shellac/alginate/bentonite system acting as an excellent cross-linker. This synergy promotes strong adhesion and creates a smoother, more compact film on the cellulose surface compared to when shellac or alginate is used individually or even as a binary shellac/alginate blend. The addition of shellac to a constant formulation of 2% sodium alginate and 1% bentonite improves the mechanical and physical properties of bagasse paper through several synergistic effects. Shellac is a natural resin with hydrophobic properties which enhances water resistance when incorporated into the composite, reducing water uptake and improving dimensional stability of the paper. Shellac forms a film that reinforces the polymer network, improving tensile strength, and stiffness of the paper. Shellac’s ester and amide bond-forming abilities enable chemical interactions with sodium alginate, further enhancing the mechanical durability^29^. Sodium alginate provides a gel-forming and binding matrix with good mechanical integrity^21^. Bentonite interacts via hydrogen bonding and electrostatic attraction with sodium alginate, increasing structural density and reducing swelling. Bentonite’s platelets intercalate in the polymer matrix to reduce water diffusion, enhancing barrier properties^30,31^.

Shellac’s film-forming and hydrophobic properties complement bentonite’s barrier effect and alginate’s gel strength, resulting in enhanced tensile strength, flexibility, and durability of the bagasse paper. This combination yields a composite with improved mechanical resistance, physical stability under moisture^21,29–31^, and better overall performance suitable for packaging or other applications.

This outcome is consistent with previous studies demonstrating that multiscale emulsion particles can coalesce due to amphiphilic oligomers driven by several forces such as heat, hydrophobic interactions, electrostatic forces, and hydrogen bonding. For example, a study developed a new bio-based wrapping material using sodium shellac; in that work, beads were produced by solidifying a complex vitamin E emulsion with sodium shellac through H⁺ and Ca2⁺ interactions followed by spray drying. Additionally, a composite film coating of shellac and gelatin was applied to prolong the shelf life of bananas^32,33^.

Furthermore, the findings here align with reports suggesting that an air permeance of around 0.001 nm/Pa is required to establish an effective oxygen barrier. It was found that cellulose nanofibers (CNF) and shellac-derived nanocomposite films provide excellent barrier properties for packaging applications^34^. On the other hand, while incorporating CNF or a CNF/shellac blend led to a slight reduction in burst strength, the overall strength remained comparable. Several factors, including the composite’s formulation, were identified as contributing to this reduction^35,36^.

The synergy within the composite arises from multiple interactions among shellac, sodium alginate, and bentonite. Shellac is a natural resin containing hydroxyl groups that can form hydrogen bonds with the carboxylate groups in sodium alginate^37^. This interaction creates an interpenetrating polymer network. The simultaneous presence of alginate not only reinforces the mechanical structure but also counteracts shellac’s inherent shortcomings (e.g., poor oxygen barrier performance). When bentonite is introduced, its layered silicate structure further enhances this network through additional physical and chemical bonding. The nanoclay’s platelet morphology increases the degree of interaction between the polymer chains and leads to more efficient cross-linking, yielding a smoother and more compact film on the cellulose surface^38^.

**Fig. 2:**
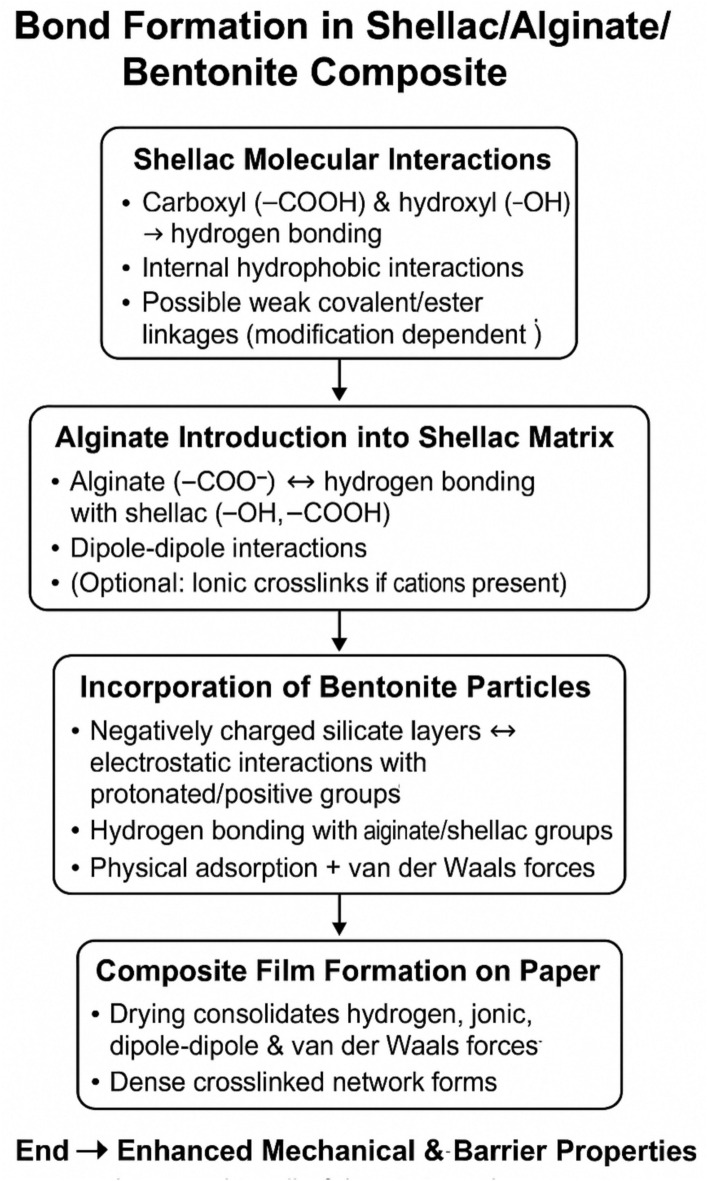

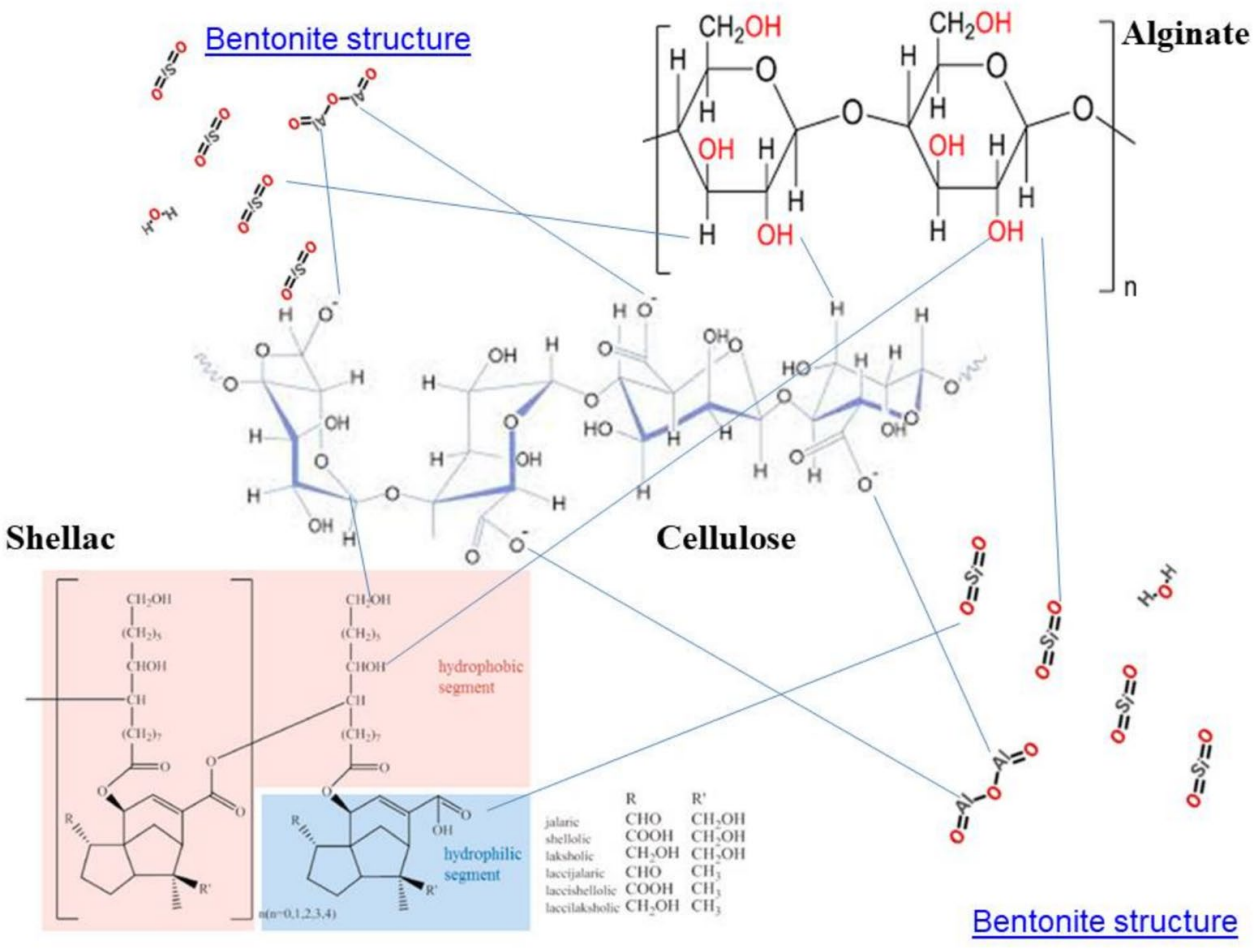
A schematic flow chart demonstrating chemical or physical bond among Shellac/Alginate/Bentonite Composite and the suggested mechanisms involving among shellac, alginate and bentonite during forming protective film layer in paper composites.

Bentonite, as a nanoclay, is especially effective at improving barrier properties^13^. When dispersed uniformly within the polymer matrix, its high aspect ratio forces gas molecules (such as oxygen) and water vapor to follow a much more tortuous (or indirect) path. This increases the diffusion distance and substantially reduces the rate at which these molecules penetrate the film. The resultant composite shows significantly improved air and water vapor permeability compared to untreated materials. The formation of a compact, cross-linked network improves not only the barrier properties but also the mechanical strengths such as burst resistance and tensile strength. The intertwined structure of shellac and alginate, reinforced by bentonite, distributes mechanical stresses more evenly throughout the matrix. The interactions between the components, including hydrogen bonding and van der Waals forces help resist deformation and failure under mechanical loads. The synergistic effect of these additives leads to a smoother, more adherent coating on the surface of the cellulose fibers. Scanning Electron Microscopy (SEM) typically reveals that the uniform dispersion of these components minimizes microstructural defects, resulting in improved surface homogeneity. This structural coherence is essential to both the mechanical integrity and the barrier performance of the final product. There are multi-mechanisms (covalent bond, hydrogen bond, dipole–dipole e.g.) as in Fig. 2 involving among shellac, alginate and bentonite during forming protective film layer in paper composites**. Also, **Fig. 2 shows a schematic flow chart demonstrating chemical or physical bond among Shellac/Alginate/Bentonite composite^21^.

### Mechanical properties

Physical and mechanical tests were conducted, including measurements of bulk density, breaking length, tensile index, Young’s modulus, tear index, and burst index, to evaluate the properties of the paper sheets. Bulk density, defined as the mass per unit volume, indicates the paper’s compactness and helps determine the relationship between its grammage (basis weight) and thickness. Paper sheets that are airy, light, opaque, and thick typically exhibit high bulk density values. Coating with shellac, bentonite, and/or alginate had a significant impact on the basis weight of the sheets. In particular, bentonite’s high density allows it to achieve bulk densities between 500 and 700 kg/m^3^, compared to approximately 400 kg/m^3^ for blank (untreated) paper.

Because basis weight and sheet thickness are directly correlated with tensile strength, these properties were compared in the study. As reported in Table 2, the bulk density of the paper decreased as the shellac content increased in samples containing 2% alginate and 1% bentonite. This trend suggests that the presence of shellac increases the spacing between the cellulose fibers, which would otherwise be tightly bonded. The optimum improvement, a 51% increase in tensile strength, was achieved using the composite formulation of 2% alginate with 1% shellac^39,40^.

**Table 2:** Improvement of mechanical properties of bagasse sheets modified with different formulations (Shellac, Alginate/Shellac, or Alginate-Bentonite/Shellac).

Sample	Bulk Density (Kg/m^3^)		Maximum Load (N)		Tensile Strength (MPa)	
	Average	% Imp.*	Average	% Imp.*	Average	% Imp.*
Blank	455.92 ± 1.26	0	42.71 ± 1.40	0	14.24 ± 0.14	0
Shellac	502.05 ± 1.47	10.12	43.68 ± 2.45	2.26	14.78 ± 0.25	3.82
SDE0	568.59 ± 1.56	24.71	59.91 ± 4.56	40.26	36.31 ± 2.76	155.03
SDE1	692.41 ± 5.74	51.87	59.70 ± 2.53	39.77	27.08 ± 0.25	90.16
SDE2	634.66 ± 2.00	39.20	76.40 ± 0.80	78.87	31.83 ± 0.08	123.58
SDE3	594.88 ± 0.00	30.48	68.57 ± 1.02	60.53	32.65 ± 0.10	129.33
SDE4	607.59 ± 5.74	33.27	76.28 ± 0.37	78.57	27.79 ± 0.04	95.16
SDE5	513.07 ± 3.21	12.53	65.15 ± 2.63	52.53	21.72 ± 0.26	52.53
LSD at 5%	57.06		8.71		3.63	
First composite: At [Alginate], 2%. Notice that: SDE0 is the paper sheet treated with alginate						
Blank	455.92	0	42.71 ±	0	14.24 ±	0
SDF0	606.21 ± 2.75	32.96	35.81 ± 0.43	− 16.17	16.24 ± 0.43	14.05
SDF1	643.52 ± 5.74	41.15	45.59 ± 6.86	6.73	19.86 ± 0.69	39.52
SDF2	586.06 ± 2.75	28.54	43.20 ± 1.82	1.14	18.82 ± 0.18	32.21
SDF3	581.89 ± 5.74	27.63	37.97 ± 0.98	− 11.10	16.13 ± 0.10	13.25
SDF4	593.36 ± 5.74	30.15	41.64 ± 1.31	− 2.52	16.62 ± 0.13	16.75
SDF5	557.94 ± 2.00	22.38	37.37 ± 6.89	− 12.52	9.78 ± 0.16	− 31.32
SDF6	526.90 ± 5.74	15.57	43.48 ± 1.80	1.79	15.84 ± 0.18	11.25
SDF7	543.56 ± 1.47	19.22	44.74 ± 1.19	4.75	14.69 ± 0.12	3.20
LSD at 5%	56.42		4.58		1.98	
Second composite: At [Bentonite], 1%. Notice: SDF0 is paper sheet treated with 1% bentonite						
Blank	455.92	0	42.71	0	14.24	0
SDG1	568.59 ± 1.56	24.71	59.91 ± 4.56	40.26	36.31 ± 2.76	155.03
SDG2	635.27 ± 2.58	39.34	62.65 ± 0.76	46.68	24.57 ± 0.08	72.56
SDG3	498.61 ± 2.68	9.36	51.79 ± 1.81	21.24	16.21 ± 0.18	13.84
SDG4	625.74 ± 1.47	37.25	53.20 ± 0.78	24.54	23.18 ± 0.08	62.79
SDG5	581.28 ± 3.56	27.50	77.07 ± 1.98	80.44	25.69 ± 0.20	80.44
SDG6	557.72 ± 1.75	22.33	74.20 ± 1.44	73.72	22.80 ± 0.14	60.11
LSD at 5%	55.98		5.83		2.28	
Third composite: Note: All samples were prepared with fixed levels of 2% sodium alginate and 1% bentonite						

*The percent improvement means how much improvement I get from the blank value.

$$\left(\text{\%}\right)Improvment=\frac{(value Treated sample-value Blank sample)}{(value Blank sample)}*100.$$

1. SDE0, SDF0 (Bagasse paper sheet treated with Alginate, 2% & Bentonite, 1%), respectively.

**Fig. 3:**
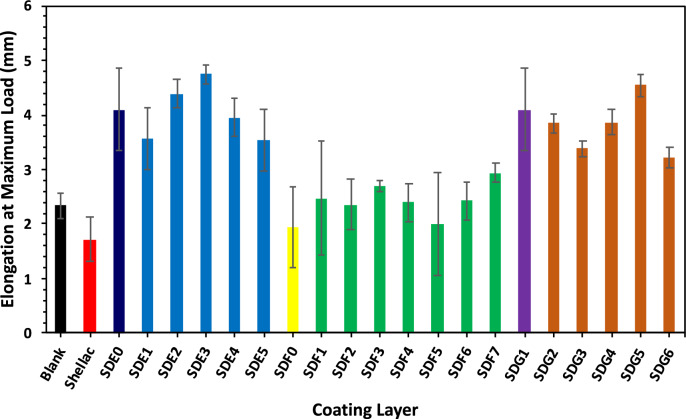
Effect of the three composite additions on elongation of bagasse sheets.

**Fig. 4:**
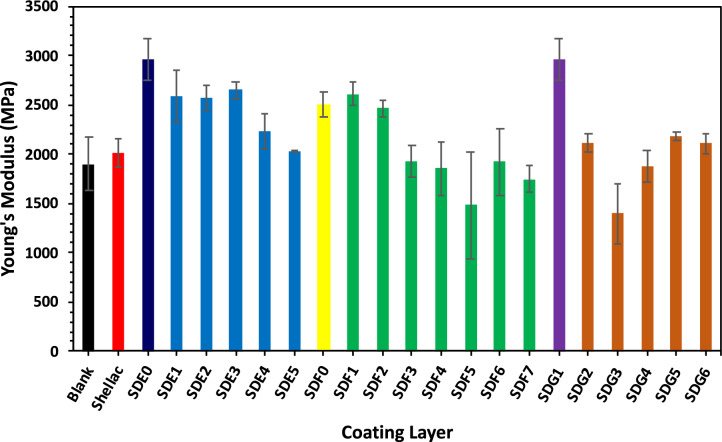
Effect of the three composite additions on Young’s modulus of bagasse sheets.

**Fig. 5:**
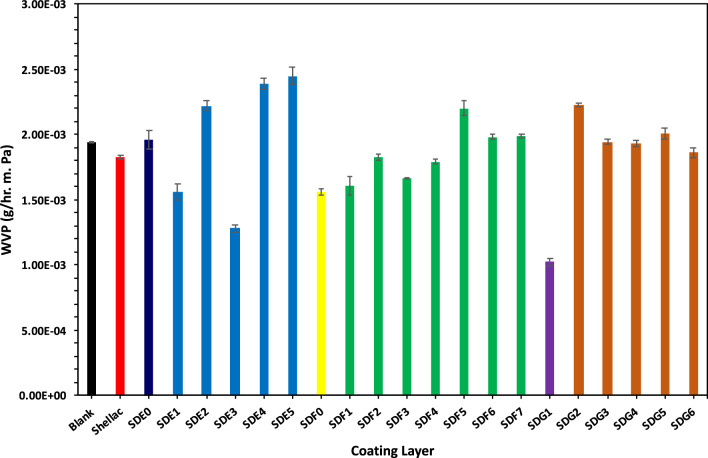
Effect of three composite additions on the WVP of bagasse sheets.

The degree of interfiber bonding and the distribution of fibers within a sheet are key factors in determining paper strength. This bonding, primarily resulting from hydrogen bonds between fibers, depends on the contact area and the inherent strength of the fibers per unit area. In general, as shown in Table 2 and Figs. 3, 4, 5, the first composite, composed of an alginate/shellac bio-nanocomposite, has a noticeable effect on the mechanical performance of the modified paper sheets. For example, increasing the shellac content up to 3% results in a significant increase in breaking length (tensile strength), after which the improvement plateaus (see Table 2). The optimum enhancement was a 51% improvement when using 2% alginate with 3% shellac. In contrast, the addition of bentonite shows only a slight effect on breaking length and tensile strength compared to using shellac or the shellac/alginate composite. Since tensile strength measures a paper’s ability to withstand breaking under tension, a property influenced by fiber strength, length, and surface area, the improvements observed here indicate a better interconnection among fibers. Notably, the combined addition of alginate with bentonite increased the breaking length by 42%.

According to Table 2, the modified paper sheets, whether treated with shellac in the presence of 2% alginate alone or together with 1% bentonite, exhibited enhanced tensile properties. In particular, the composite of 2% alginate with 3% shellac achieved an optimum improvement of 129% in tensile strength when compared with untreated paper. The presence of an alginate coating notably strengthens the paper, as shellac reacts with hydroxyl groups on the paper surface to enhance viscoelastic properties^37^. This synergistic effect, in combination with either alginate alone or alginate with bentonite, improves overall mechanical performance. In addition, modified papers with higher basis weights demonstrated better strength and elongation. However, it was observed that the tensile strength may decrease with an increase in nanoclay content, likely due to alterations in the adhesion between the base paper and the hybrid layer.

Studies on composite materials indicate that the bonding between components significantly influences a composite’s overall mechanical properties. The maximum percentage improvements achieved with a formulation of 2% alginate and 3% shellac were 52% for breaking length, 103% for elongation at maximum load, 129% for tensile strength, 116% for tensile index, and 40% for Young’s modulus.

- Here’s why adding shellac to alginate, bentonite, or their mixture increases tensile strength compared to untreated paper:
  - Shellac forms strong cross-links with the polymer chains of alginate or bentonite, creating a compact and cohesive network.
  - This network improves film uniformity and adhesion to the paper fibers, enhancing mechanical strength^29,30^.
  - The combination reduces voids and defects, making the paper surface more resistant to tension and tearing.
- But, here’s a clear explanation about how shellac affects tensile strength and modulus:
- Shellac increases tensile strength by forming strong cross-links with polymers like alginate or bentonite, creating a compact network that resists breaking under tension.
- At the same time, it lowers the modulus (stiffness) because the film becomes less rigid and more flexible. Shellac’s natural resinous nature adds a bit of elasticity, allowing the material to stretch more before breaking^29^.
- So, the material becomes stronger but less brittle, improving durability while reducing stiffness.

The composite additives also improved the elongation of the paper sheets (Fig. 3) and Young’s modulus (Fig. 4), indicating enhanced ductility resulting from the optimized interaction of shellac, alginate, and bentonite in the coating. The 129% increase in tensile strength and 103% in elongation at break are highly competitive and often exceed values for commercial biodegradable coatings, especially those using single biopolymer systems., including CNF/shellac films^29^.

The air and water vapor barrier properties of the shellac-, alginate-, and/or bentonite- modified paper sheets were also evaluated. Air and water vapor, which can compromise packaged goods by promoting microbial growth and causing degradation, must be effectively blocked in packaging applications. Compared to untreated paper, the modified samples exhibited a significant reduction in air permeability (Table 1) and WVP (Fig. 5).

Moisture negatively affects shelf life by accelerating autoxidation, vitamin degradation, enzymatic reactions, and microbial growth, as well as altering texture and crispness^41^. Thus, low water vapor transmission rates (WVTR) are highly desirable. The water vapor permeability (WVP) of the coatings decreased with increasing percentages of bentonite and alginate, likely due to the accumulation of suspended particles that retard moisture transfer. A maximum improvement in WVP of 47.36% was achieved with a coating composed of 2% alginate and 1% bentonite. Moreover, the presence of shellac further improved the water barrier properties of the composite. In fact, as the percentage of shellac in the coating increased, the water barrier properties also improved, for instance, a 3% shellac coating combined with 2% alginate reduced the WVP by up to 35% compared to untreated paper.

This enhanced barrier effect is explained by the formation of a well-organized network within the coating. The cross-links between shellac, alginate, and bentonite create a tortuous path that restricts the passage of air and water vapor, thereby substantially reducing permeability. However, increasing the shellac concentration from 1.0% to 3.0% may lead to the agglomeration of shellac particles within the 2% alginate matrix, which can create open spaces and slightly increase WVP^42^, this increase is more pronounced at higher shellac concentration (4 & 5%)^29^.

The oil resistance of the paper coatings containing shellac, bentonite, and/or alginate was superior to that of the control paper, as evidenced by the complete absence of oil penetration in all the samples tested. All modified papers demonstrated high resistance to oil penetration, attributable to the high hydrophobicity of the coating layer formed by shellac combined with alginate and/or bentonite^43,44^.

### X-ray diffraction analysis

 Typically, the effects of bentonite on paper sheets are evaluated in terms of their mechanical and barrier properties, while the potential impact of crystallographic conformation; such as the crystallinity index, apparent crystallite size, and interlayer distance of the cellulose fibers; is often overlooked in relation to the final physicomechanical the final physicomechanical characteristics. To address this, XRD analysis was performed to study the influence of shellac, alginate, and/or bentonite treatments on the structural properties of the paper sheets.

**Fig. 6:**
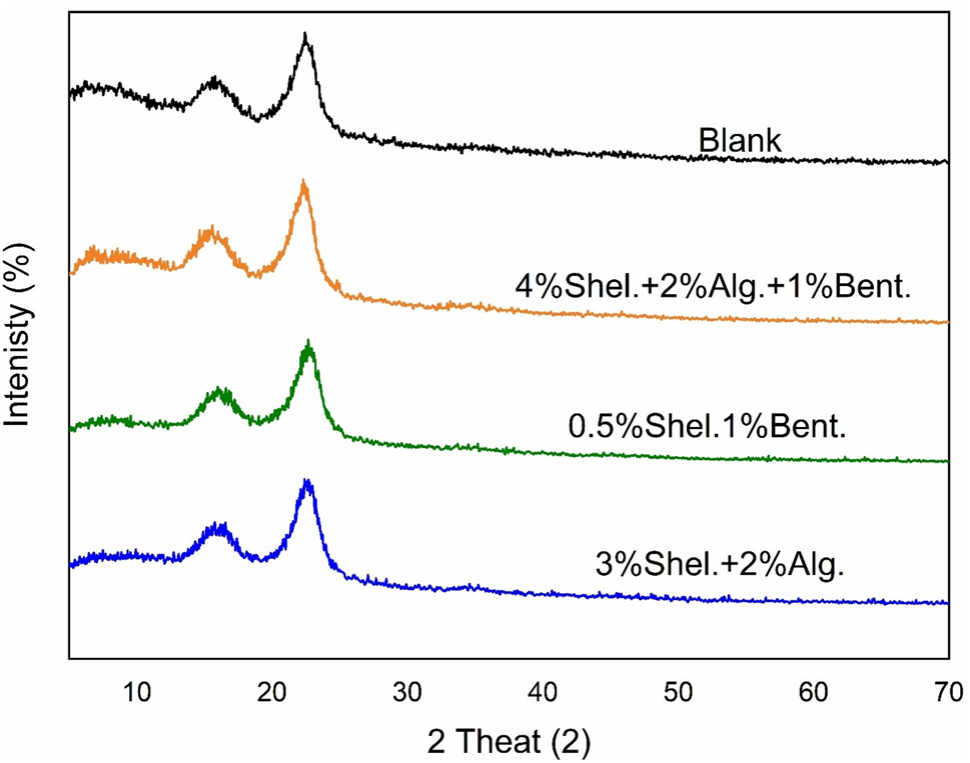
XRD analysis for different sheets modified with various composites.

Figure 6 displays the XRD patterns for untreated paper sheets and those treated with 3% shellac in the presence of 2% alginate and 1% bentonite. Because the X-ray penetration depth was much smaller than the thickness of the shellac layer, the XRD traces primarily represent the structure of shellac. The pattern exhibited two prominent peaks at 15.20° 2θ and 22.10° 2θ. Changes in the intensity of these peaks can be attributed to alterations in the molecular structure of the resin during polymerization, suggesting that shellac may enhance the stability of the coating^43^.

### Thermogravimetric analysis (TGA)

**Fig. 7:**
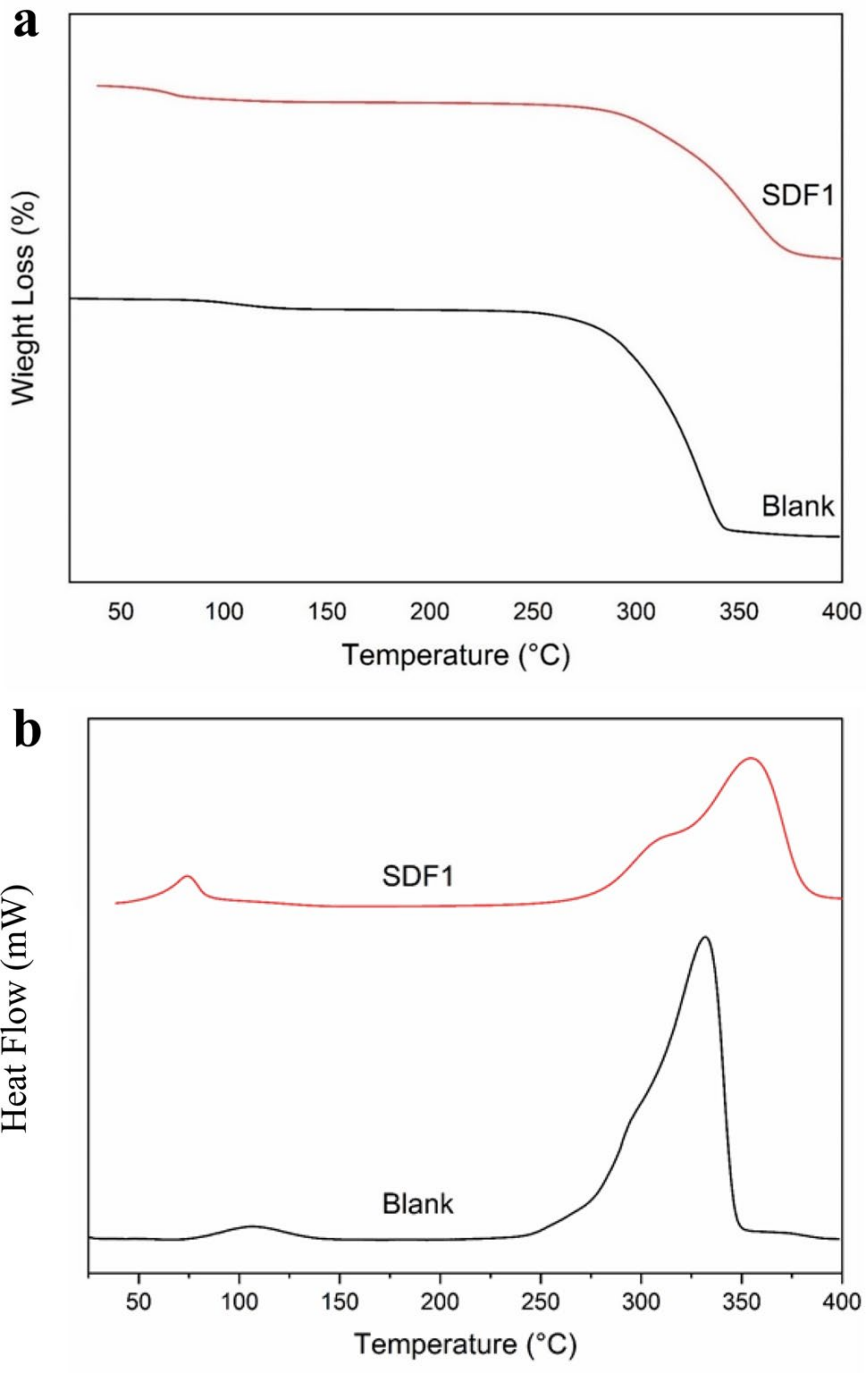
(**a**) TGA analysis of blank bagasse sheets and bentonite-modified sheets. (**b**) DSC analysis for blank bagasse sheets and bentonite-modified sheets.

As shown in Fig. 7a, the sample’s weight was measured during thermogravimetric analysis (TGA), which is useful for examining the thermal stability and degradation behavior of materials for various practical applications. DSC analysis for blank bagasse sheets and bentonite-modified sheets were shown in Fig. 7b. It was observed that the shellac-modified paper (0.5%) did not exhibit any appreciable weight loss due to moisture at temperatures below 57–75 °C. The shellac- modified paper began to decompose at a start decomposition temperature (Td) of 290 °C. However, the glass transition (Tg) was visible up to 354 °C as in Fig. 7b, with substantial weight loss occurring between 304 and 354 °C. This stage is ascribed to the decomposition, deacetylation, and depolymerization of the organic hydrocarbon backbone of the shellac segment^23^.

#### Scanning electron microscopy (SEM) of paper sheet coatings with shellac/bentonite and/or sodium alginate films

**Fig. 8:**
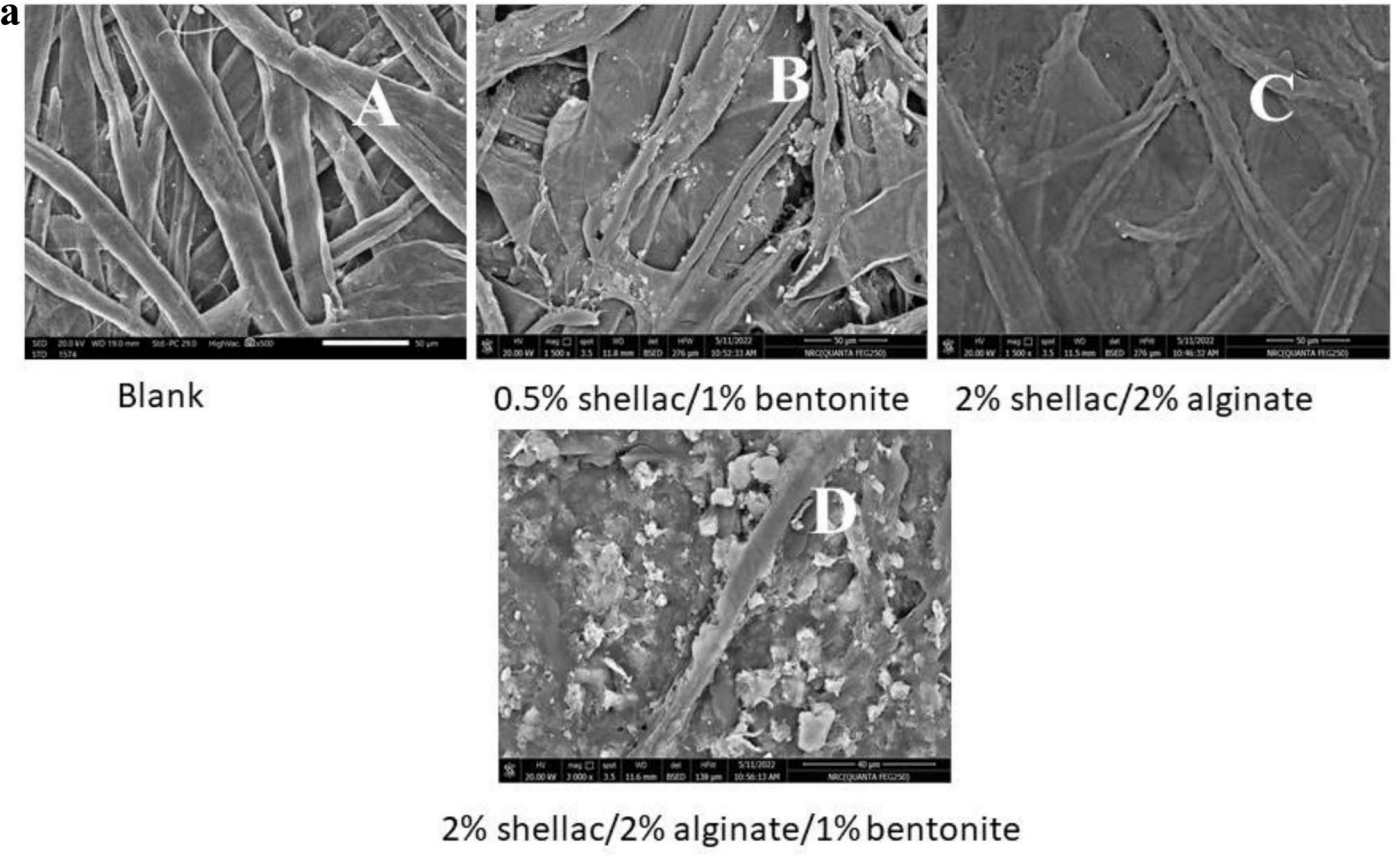

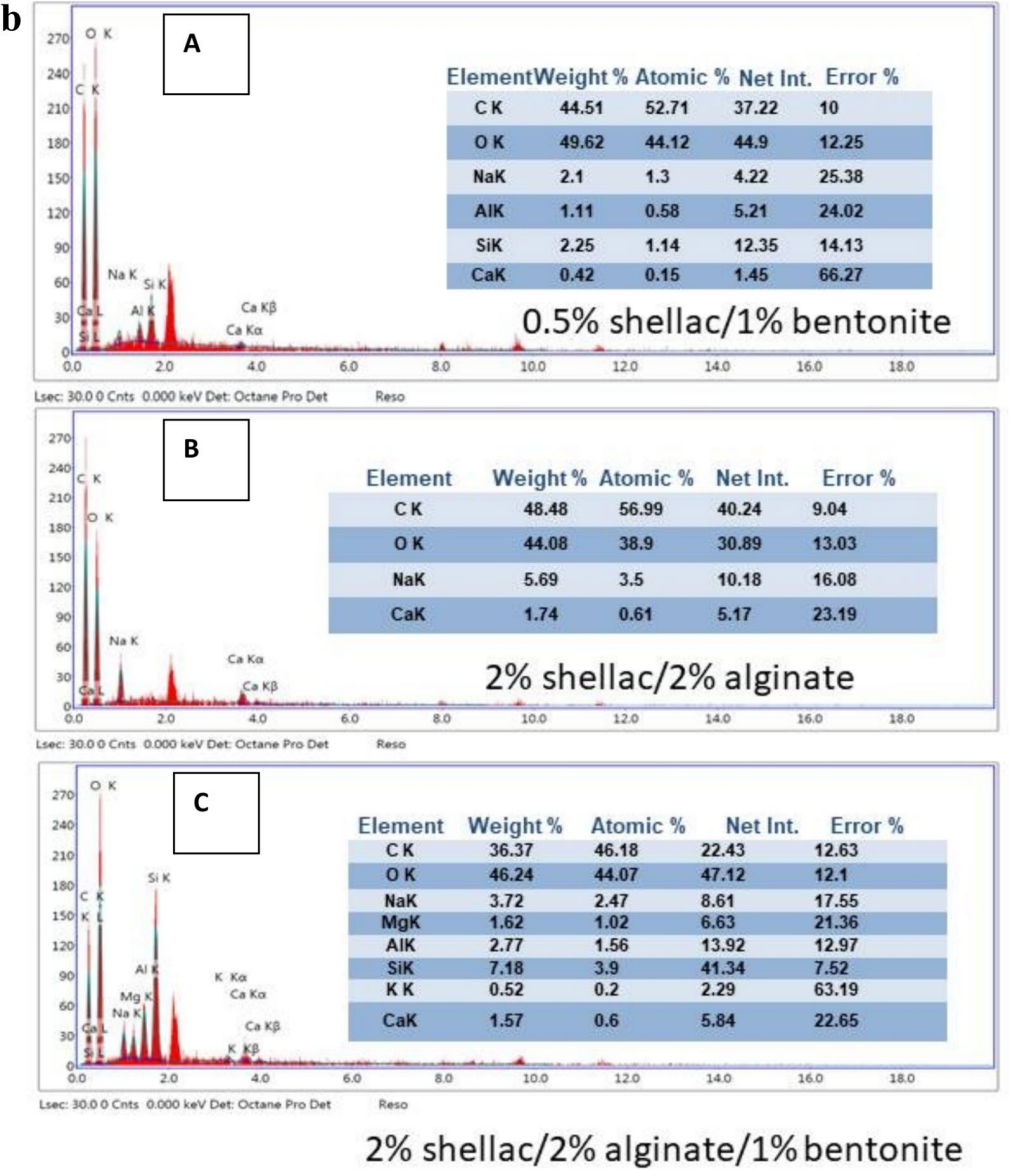
(**a**) SEM images of paper sheet surfaces: (**A**) Untreated, (**B**) Modified with 0.5% shellac/1% bentonite, (**C**) Modified with 2% shellac/2% alginate, and (**D**) Modified with 2% shellac/2% alginate/1% bentonite. (**b**) EDX spectra of paper sheet surfaces: (**A**) Untreated, (**B**) Modified with 0.5% shellac/1% bentonite, (**C**) Modified with 2% shellac/2% alginate, and (**D**) Modified with 2% shellac/2% alginate/1% bentonite.

SEM was used to examine the surface morphology of paper sheets modified with three different formulations, shellac/bentonite, shellac/sodium alginate, and shellac/bentonite/sodium alginate mixture. Figure 8a illustrates the film homogeneity. The samples analyzed were films on paper sheets; (A) untreated (blank), (B) modified with 0.5% shellac/1% bentonite and (C) modified with 2% shellac/2% alginate, and (D) 2% shellac/2% alginate/1% bentonite.

These samples were characterized by SEM (Fig. 8a) and were chosen based on their superior mechanical and physical properties. The SEM images confirm good film formation for all coatings. In particular, composite films of alginate and shellac are evident on and between the paper sheet fibers. However, differences in pore structure were observed among the samples; the 0.5% shellac/1% bentonite coating still exhibited small pores, whereas the coatings containing 2% shellac/2% alginate; with or without 1% bentonite; displayed smoother, better-covered surfaces. This indicates that the presence of sodium alginate mixed with shellac (with or without bentonite) is crucial as a cross-linker, enhancing the interaction between the cellulose OH groups and the COO⁻, C=O, and OH groups of shellac, as well as with the material components of bentonite (silica (SiO₂) and alumina (Al₂O₃))^45^, Which was confirmed to be present in films in different proportions through EDX spectra analysis as in Fig. 8a,b.

These connections arise from chemical bonds, van der Waals forces, and hydrogen bonds. Hydrogen bonds result from dipole–dipole attractions (i.e., between strongly electronegative atoms such as oxygen or nitrogen bound to hydrogen), while van der Waals forces, although weaker and highly distance-dependent; also contribute to the molecular adhesion^46^. At greater separations, van der Waals forces rapidly diminish. Overall, these findings corroborate the significant improvements in air permeability, tensile strength, and burst strength observed in the treated paper compared to untreated paper.

In summary, the robust interaction between shellac, alginate, and bentonite; mediated by hydrogen bonds, physical cross-linking, and enhanced tortuosity effects; results in a unique composite with superior barrier and mechanical properties. These properties not only make the material suitable for food packaging but also open up possibilities for its use in a variety of environmentally friendly and sustainable applications by addressing many drawbacks of conventional petroleum-based packaging.

### Measurement of contact angle

**Fig. 9:**
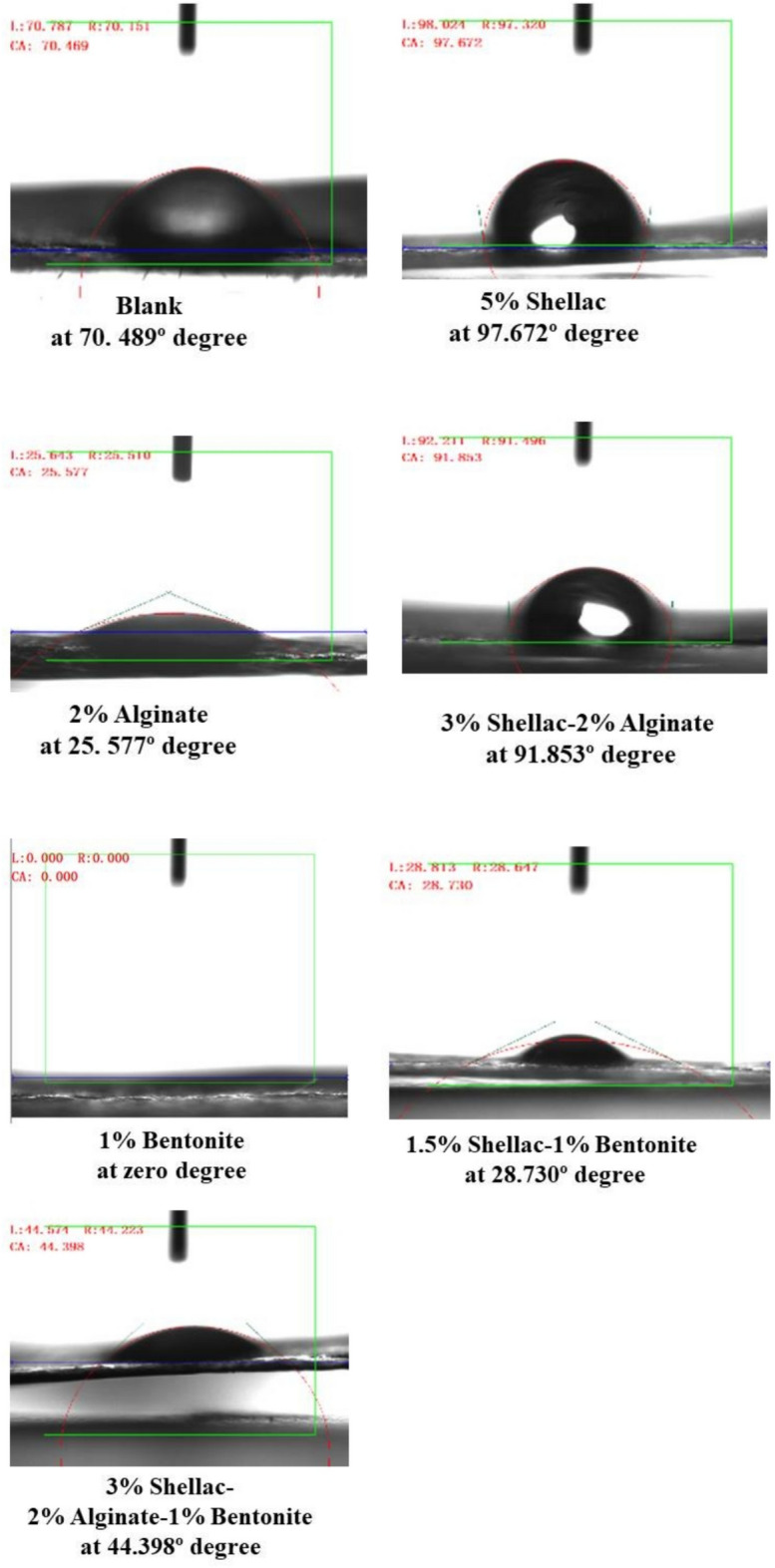
The contact angles of the modified paper sheets (wettability).

To assess surface wettability, static water contact angles were measured by spraying water droplets onto the modified paper sheets. The untreated paper (Blank) exhibited a moderate hydrophilicity with a contact angle of 70.47° (Fig. 9). Upon coating with shellac—a hydrophobic natural resin which lowering surface energy, the contact angle increased to 97.67°, indicating strong hydrophobicity and superior water resistance^16^.

In contrast, treating the paper with alginate or bentonite alone drastically reduced the contact angle to 25.57° and 0°, respectively, reflecting their superhydrophilic nature. The zero contact angle (0.00°) observed for the 2% bentonite sample is primarily attributed to the inherent hydrophilic nature of bentonite clay, which strongly absorbs water due to its layered structure and charged sites attracting water molecules^30^. Bentonite’s surface chemistry promotes water spreading and absorption rather than droplet formation. Regarding the contrast with the blank paper (contact angle ~ 70.5°), the blank paper surface is less hydrophilic, showing more water repellency than bentonite. Bentonite particles can alter the composite’s surface morphology, introduce micro- and nano-scale roughness or reduce porosity which increase the contact angle^30,31^.

However, when shellac was combined with these hydrophilic materials, the contact angle increased significantly from 0° (bentonite alone) to 28.73° with shellac–bentonite, and from 25.57° (alginate alone) to 91.85° with shellac–alginate which nearly matched pure shellac. These findings confirm that the addition of shellac enhances surface hydrophobicity, even in the presence of highly hydrophilic materials. The ternary mixture (shellac–alginate–bentonite) yielded an intermediate contact angle of 44.40°, reflecting the balance between hydrophobic and hydrophilic components.

The highest contact angles—and thus greatest hydrophobicity—were achieved with 5% shellac (97.67°) and 3% shellac–2% alginate (91.85°), representing increases of 38.5% and 30.3%, respectively, compared to the untreated sample. These results suggest that in binary or ternary coatings, sufficient shellac content can dominate and mask the hydrophilicity of alginate or bentonite, significantly improves the paper’s resistance to water. This enhanced barrier performance aligns with other analytical findings from tensile strength, burst strength, air permeability, SEM, and TGA measurements, supporting the effectiveness of the coating strategies used^1,21,47^.

Contact angle values exceeds 90° in our best formulations indicate enhanced hydrophobicity that is on par with commercial coatings like Alkyl Ketene Dimer (AKD) or wax emulsions^48^. A comparison between contact angle and WVP values for the investigated samples emphasizes how increasing surface hydrophobicity enhances moisture‐barrier performance. Figure 9 illustrate the contact angles of the modified samples, highlighting changes in surface wettability and confirming the improved moisture-barrier properties.

### The antibacterial properties of treated bagasse paper sheets

The antibacterial activity of the treated samples SDE(1–5), SDF(0–7) and SDG1-5, against Gram-negative bacteria Escherichia coli, and Gram-positive bacteria ***Bacillus subtilis* (B)** as in Table 3. The antimicrobial properties of shellac^1,16^; combined with the strong binding of the bio-additives to the alginate and/or bentonite blend (which prevents their leaching and the formation of inhibition zones); account for the lack of observed inhibition zones and microbial growth in all treated samples, in contrast to the blank sample. SEM images further indicate that shellac, alginate, and/or bentonite are firmly bonded to the bagasse paper matrix, forming robust, adherent macromolecular networks on the surface. Incorporating shellac into these formulations enhances the treated paper’s antibacterial capabilities against both bacterial strains^49^.

**Table 3:** Displays the antibacterial activity of the treated samples SDE(0–5), SDF(0–7) and SDG(1–5).

No. of samples	5%Shellac (wt.%)	Observations	
		Gram-positive	Gram-negative
		*Bacillus subtilis* (B)	*Escherichia coli* (E)
Blank	0.0	Bacterial growth	Bacterial growth
SDE0	0.0	Bacterial growth	Bacterial growth
SDE1	1.0	No bacterial growth	No bacterial growth
SDE2	2.0	No bacterial growth	No bacterial growth
SDE3	3.0	No bacterial growth	No bacterial growth
SDE4	4.0	No bacterial growth	No bacterial growth
SDE5	5.0	No bacterial growth	No bacterial growth
At [Alginate], 2%			
SDF0	0.0	No bacterial growth	No bacterial growth
SDF1	0.5	No bacterial growth	No bacterial growth
SDF2	1.0	No bacterial growth	No bacterial growth
SDF3	1.5	No bacterial growth	No bacterial growth
SDF4	2.0	No bacterial growth	No bacterial growth
SDF5	2.5	No bacterial growth	No bacterial growth
SDF6	3.0	No bacterial growth	No bacterial growth
SDF7	3.5	No bacterial growth	No bacterial growth
At [Bentonite], 1%			
SDG1	1.0	No bacterial growth	No bacterial growth
SDG2	2.0	No bacterial growth	No bacterial growth
SDG3	3.0	No bacterial growth	No bacterial growth
SDG4	4.0	No bacterial growth	No bacterial growth
SDG5	5.0	No bacterial growth	No bacterial growth
At [Alginate], 2%/ [Bentonite], 1%

**Table 4:** The mechanical, physical, and performance characteristics of the composites in previous published studies.

Film composites	The most important results	Ref
Dalla Rosa, Biodegradable polymers for food packaging: a review Cellulose Nanofiber/Shellac Nanocomposite Films As Coatings For Packaging Paper	Air permeability: The treated paper sheets showed no measurable air, which is comparable to or better than values reported for conventional polyethylene-coated paperboard and bio-based coatings	^28,29^
Moisture barrier, wetting and mechanical properties of shellac/agar or shellac/cassava starch bilayer bio-membrane for food applications	Contact angle and hydrophobicity: Contact angle values reaches almost 90° in our best formulations indicate enhanced hydrophobicity that is on par with commercial coatings like AKD or wax	^48^
Cellulose Nanofiber/Shellac Nanocomposite Films As Coatings For Packaging Paper	Mechanical strength: The 129% increase in tensile strength and 103% in elongation at break are highly competitive and often exceed values for commercial biodegradable coatings, especially those using single biopolymer systems., including CNF/shellac films	^29^
Edible Oleoresin Infused Nanocomposite Film: A Novel Strategy for Nut Preservation and Aflatoxin Control’ has been accepted for publication in Food Biophysics	In order to improve nut preservation and reduce aflatoxin contamination, this study investigates the application of coatings made of chili and garlic oleoresin nanoparticles. The findings showed that garlic oleoresin increased the mechanical resilience of alginate membranes from 6.05 MPa to 27.18 MPa, while chili oleoresin increased the tensile strength of maltodextrin-based membranes from 1.71 MPa to 13.99 MPa SEM structural analysis verified modifications in membrane architecture brought about by the addition of oleoresin. By extending induction durations by 23.1 h for hazelnut oil and 10.4 h for almond oil in comparison to controls, chili oleoresin nanoparticles improved the oxidative stability of nut oils. In experimentally inoculated peanut samples, nanoemulsified chili oleoresin coatings reduced aflatoxin contamination by 100%, while garlic nanoemulsion decreased aflatoxin levels by 87.97%	^7^
Preparation of Antibacterial Cellulose of Non-Woven Cotton Fabric Treated with Curcumin/Shellac Based on Polyvinyl Alcohol/Sodium Caseinate Blends for Potential Packaging Purposes	When PVA/SC/curcumin/shellac formulation was applied to the NWC fabric sample, the following outcomes were observed: a) decreased swelling characteristics combined with an increase in the treated fabric’s gel fraction b) Increased antimicrobial activity of the treated fabric against filamentous fungus (Aspergillus niger), pathogenic yeast (Candida albicans), and Gram-positive and Gram-negative bacteria (Staphylococcus aureus and Escherichia coli); c) Reduced air permeability combined with an increase in the fabric’s tensile, Young’s, and burst strengths; and d) The best water vapor transmission rate when compared to other treated formulations. In comparison to untreated non-woven, the addition of PVA/SC as in (NW1, 105.05°) and PVA/SC/CUR/SH as in (NW9, 124.31°) resulted in maximum hydrophobicity values that improved the contact angle of NWC by 1219.28 and 1461.13%, respectively	^16^

These data in Table 4 confirm that our ternary shellac/alginate/bentonite coating not only performs on par with, but in several cases exceeds, the barrier and mechanical standards of both current bio-based coatings and traditional commercial counterparts—highlighting its strong potential for eco-friendly packaging applications^51^.

Paper-based packaging has emerged as a more sustainable alternative to plastics. Bagasse paper, a low-cost and renewable byproduct of sugarcane processing, holds significant potential for sustainable packaging and adds a layer of environmental and economic relevance. However, its poor resistance to water, oil, and gas permeability, inadequate mechanical strength, and limited antimicrobial activity restrict its application range. To overcome these limitations, functional coatings based on biodegradable polymers and natural materials have been investigated to enhance the performance of paper substrates^29,50^.

### Limitations and future perspectives

While the results of this study demonstrate significant improvements in the mechanical and barrier properties of bagasse paper sheets using a shellac/alginate/bentonite composite coating, several potential limitations must be acknowledged. First, the durability of the coating under mechanical stress, abrasion, or prolonged use has yet to be fully assessed. Extended fatigue and abrasion testing, as well as chemical cross-linking to improve wear resistance, represent important next steps. Second, although the individual coating components—shellac, alginate, and bentonite—are generally recognized as safe and used in food-related applications, formal migration and sensory evaluations as well as compliance with food-contact regulations are needed to confirm suitability for direct food contact. Third, the impact of the coating on recyclability of the paper substrate has not been evaluated; coatings can affect fiber recovery during repulping and may alter the biodegradation rate depending on coating thickness and formulation. Finally, since alginate and bentonite are hydrophilic in nature, performance under high humidity conditions may be reduced, despite shellac’s contribution to surface hydrophobicity. Long-term exposure to moisture and cyclic humidity testing should therefore be included in future studies to assess barrier stability and coating integrity under realistic storage conditions.

## Conclusion

This study investigated the effects of varying shellac content on the mechanical, hydrophobic, and air-permeability properties of bagasse paper sheets modified with bentonite and alginate. Three composite formulations were prepared and applied as barrier-enhancing coatings for paper-based packaging materials. The first composite involved 2% alginate solution mixed with (1–5%) shellac; the second composite involved mixing 1% bentonite solution with (0.5–3.5%) shellac; and the third composite involved combining 1% Bentonite/2% alginate with (1–5%) shellac. These composite solutions were applied using a coating technique. SEM analysis confirmed the uniform distribution of shellac with sodium alginate and/or bentonite throughout the paper sheets. Key parameters such as thickness, air permeability, tensile strength, burst strength, and Young’s modulus was evaluated.

The results demonstrated that incorporating bentonite and alginate in a shellac matrix significantly improved the mechanical and physical properties of the paper sheets. Notably, when 2% shellac was used, the modified sheets exhibited no measurable air permeability (compared to 31.15 s for untreated sheets) and burst strength increased by 104.65% and 100%, respectively, reaching 4.4 and 4.3 kg/cm2, when combined with 2% alginate or with 2% alginate/1% bentonite, compared with 2.15 kg/cm2 for untreated paper. The maximum improvements in mechanical properties were achieved with 2% alginate combined with 3% shellac, registering enhancements of 52% in breaking length, 103% in elongation at maximum load, 129% in tensile strength, 116% in tensile index, and 40% in Young’s modulus.

Overall, compared to untreated bagasse paper sheets, the modified sheets exhibit excellent improvements in tensile strength, air permeability, water vapor permeability (WVP), thermal stability, and antibacterial activity. These findings indicate that bagasse paper sheets loaded with a shellac/alginate-bentonite composite have considerable potential as future packaging materials in environmentally friendly and sustainable applications.

## Data Availability

All data generated or analysed during this study are included in this published article.
